# Comprehensive Analysis of Prognostic Value and Immune Infiltration of TFAP2 Family Members in Bladder Cancer from Database and FFPE Sample

**DOI:** 10.7150/jca.86838

**Published:** 2023-09-18

**Authors:** Feng Yuan, Yi Sun, Guang-Cheng Dai, Qiu Yao, Yi-bing Zhou, Ya-cheng Zang, Xiao-Long Liu, Bo-Xin Xue

**Affiliations:** 1 Department of Urology, The Second Affiliated Hospital of Soochow University, Suzhou, China.; 2 School of Biology and Basic Medical Sciences, Medical College of Soochow University, Suzhou, China.

**Keywords:** TFAP2 family, bladder cancer, prognosis, immunes infiltration, TAM polarization

## Abstract

**Background:** Bladder cancer (BLCA) is one of the common malignant tumors worldwide. Recent studies have shown that Transcription factor activating protein-2(TFAP2) family proteins plays a bidirectional regulatory role in the process of tumorigenesis versus evolution by regulating the expression of tumor associated genes. However, little is known about the function of distinct TFAP2s proteins in patient with BLCA.

**Methods:** Formalin-fixed paraffin-embedded (FFPE) sample tissues and clinical data of 240 patients with bladder cancer were collected for immunohistochemical analysis. The Human Protein Atlas, Gene Expression Profiling Interactive Analysis (GEPIA), Shiny Methylation Analysis Resource Tool (SMART), Kaplan-Meier plotter, cBioPortal, Metascape, LinkedOmics, TIMER and CIBERSORT were utilized to analyze differential expression, prognostic value, genetic alteration and immune cell infiltration of TFAP2 family in patients with BLCA.

**Results:** Our study found that TFAP2 family proteins are generally expressed higher in BLCA tissues than in normal tissues. However, they show different trends in the growth, metastasis and survival prognosis of BLCA. TFAP2A and TFAP2C was associated with worse clinical stage and prognosis in BLCA patients, while TFAP2B, TFAP2D and TFAP2E showed the opposite trend. Importantly, the functions of the differentially expressed TFAP2s were primarily related to the developmental process, reproductive process, response to stimulus and immune system process, etc. Moreover, TFAP2 family was significantly correlated with the infiltration of six immune cell types and might regulate TAM polarization.

**Conclusion:** TFAP2 family might be an important regulator of immune cell infiltration and a valuable prognostic biomarker in patients with BLCA.

## Introduction

Transcription factor activating protein-2(TFAP2) is a family of transcription factors with cell type specific DNA binding, and activated genes are selectively expressed and participate in a series of cellular life activities, such as the regulation of cell proliferation, differentiation, and apoptosis 1. TFAP2 also plays an important role during embryonic development in mammals 2. Recent studies have shown that TFAP2 plays a bidirectional regulatory role in the process of tumorigenesis versus evolution by regulating the expression of tumor associated genes 3-5. Today, there are five TFAP2 family members that have been identified in humans, namely TFAP2A, TFAP2B, TFAP2C, TFAP2D and TFAP2E.

Previous studies have shown that TFAP2A and TFAP2C have inhibitory effects on tumor growth in a variety of tumors 6-8. However, TFAP2A and TFAP2C is highly expressed in bladder cancer, lung cancer and other tumors, and overexpression of TFAP2A and TFAP2C is associated with poor prognosis of many tumors. Recent study has shown that TFAP2A can inhibit the proliferation of lung cancer cells while overexpressing TFAP2A can promote tumor metastasis 5. In BLCA, TFAP2A overexpression decreased tumor cell viability, migratory potential, while TFAP2C shows the opposite trend 9. A further study revealed that TFAP2A and TFAP2C overexpression cooperate with other TFs to promote the basal-squamous transition during BLCA progression 10. Therefore, the role of TFAP2 protein family in tumors is complex, and its integral function in tumors needs further exploration.

Bladder cancer (BLCA) is the most diagnosed cancer in the urinary system. In recent years, BLCA cause of incidence and deaths rate are increased gradually, as well as more than 440,000 new cases and 130,000 deaths one year 11. At present, the comprehensive treatment and management of BLCA patients are still not optimistic, and the prognosis is poor 12. Therefore, we extended the research field to BLCA based on a variety of large databases, with the purpose of determining the potential oncogenic values of distinct TFAP2 family members in BLCA.

## Materials and methods

### Clinical data and pathological specimens

A total of 240 formalin-fixed paraffin-embedded (FFPE) BLCA tissues samples and 36 adjacent normal tissues samples were collected from patients diagnosed with BLCA at The Second Affiliated Hospital of Soochow University. The study was conducted in accordance with the Declaration of Helsinki (as revised in 2013). This study was approved by ethical committee of the Second Affiliated Hospital of Soochow University (ethical code: JD-LK-2019-104-01). The inclusion criteria were as follows: patients underwent transurethral resection or radical bladder resection, the postoperative pathology was urothelial carcinoma, patients should undergo CT screening before surgery to facilitate comprehensive clinical staging. Exclusion criteria: the specimen removed is insufficient for the next research, patients with other cancer, patients with severe comorbidities, such as heart failure, serious liver or kidney dysfunction.

### Immunohistochemistry

Immunohistochemical staining was performed at the Department of Pathology in The Second Affiliated Hospital of Soochow University. The tumors were fixed in 4% paraformaldehyde, embedded in paraffin, sectioned, and then stained with 1,2-dibromoethane-conjugated antibody (Abcam). Firstly, these sections were deparaffinized and rehydrated. Afterwards, we performed antigen retrieval in antigen retrieval solution using a steamer autoclave at 121℃ for 15 minutes. Nonspecific proteins were blocked with 5% BSA for 30 minutes, and sections were then rinsed with 0.01 mol/L PBS for 3 minutes. The sections were then respectively incubated with the detection antibody overnight at 4°C. Afterward, the sections were incubated with secondary antibody at room temperature for 1 hour and followed by DAB (DAKO Liquid DAB) staining for 5 min. Finally, hematoxylin counterstain was used to show the cellular nucleus. After the sections were sealed with neutral balsam, the whole images were taken and analyzed. The intensity of fluorescence in them was measured by ImageJ from at least 3 sections.

### GEPIA

GEPIA (http://gepia.cancer-pku.cn/) is a newly developed interactive web server for analyzing the RNA sequencing expression data of 9,736 tumors and 8,587 normal samples from the TCGA and the GTEx projects, using a standard processing pipeline. In this study, GEPIA provides functions such as tumor/normal differential expression analysis, profiling according to pathological stages, patient survival analysis.

### Shiny Methylation Analysis Resource Tool (SMART)

SMART (http://www.bioinfo-zs.com/smartapp/) is an interactive web server for analyzing DNA methylation of TCGA project. We used it to compare the methylation of TFAP2 family genes in BLCA and adjacent tissues, and to analyze the correlation between TFAP2 family protein methylation and pathological staging of BLCA.

### The human protein atlas

The Human Protein Atlas (https://www.proteinatlas.org/) is a Swedish-based program initiated in 2003 with the aim to map all the human proteins in cells, tissues, and organs using an integration of various omics technologies, including antibody-based imaging, mass spectrometry-based proteomics, transcriptomics, and systems biology.

### Kaplan-Meier Plotter

The Kaplan Meier plotter (https://kmplot.com/analysis/) is capable to assess the correlation between the expression of all genes (mRNA, miRNA, protein) and survival in 30k+ samples from 21 tumor types including breast, ovarian, lung, & gastric cancer. Sources for the databases include GEO, EGA, and TCGA.

### cBioPortal

The cBioPortal (http://www.cbioportal.org/) website integrates data from 126 tumor genome studies, including large tumor research projects such as TCGA and ICGC, and covers data from 28000 samples. In addition, some samples also include phenotypic information such as clinical prognosis. Based on The Cancer Genome Atlas (TCGA) database, genetic alterations, and the network module of TFAP2 were obtained from cBioPortal.

### String

The STRING website (https://string-db.org/) integrated and constructed the PPI using computational predictions, which visualized the potential proteins interacting with TFAP2 genes.

### Metascape

Metascape (http://metascape.org) integrates more than 40 gene function annotation databases and supplies various visualization methods, allowing readily gene function analysis. Herein, we employed this database to perform enrichment analysis on TFAP2 related genes obtained from String database. The analysis included gene ontology (GO) and Kyoto Encyclopedia of Genes and Genomes (KEGG) enrichment analysis. We set min overlap as 3, min enrichment as 1.5, and P 0.05 as significant.

### LinkedOmics

LinkedOmics (http://www.linkedomics.org/login.php) is publicly available portal that includes multi-omics data from all 32 TCGA Cancer types and 10 Clinical Prote-omics Tumor Analysis Consortium (CPTAC) cancer cohorts. The web application has three analytical modules: LinkFinder, LinkInterpreter and LinkCompare. LinkFinder allows users to search for attributes that are associated with a query attribute. To de-rive biological insights from the association results, the LinkInterpreter module per-forms enrichment analysis based on Gene Ontology, biological pathways, network modules, among other functional categories.

### Timer

TIMER web server is a comprehensive resource for systematical analysis of immune infiltrates across diverse cancer types. The abundances of six immune infiltrates (B cells, CD4+ T cells, CD8+ T cells, Neutrophils, Macrophages, and Dendritic cells) are estimated by TIMER algorithm. We evaluated the relationship between the level of TFAP2 expression and the abundance of six types of TIICs above.

### CIBERSORT

Transcriptomic profiles of BLCA patients from TCGA database (https://xenabrowser.net or https://portal.gdc.cancer.gov) were download and cibersort was used to elucidate the correlation between TFAP2s and immune cells infiltration. All data were analyzed with R platform 4.1.2 (http://www.r-project.org).

### Statistical Analysis

The associations of the expression of TFAP2s with clinicopathological data were evaluated by the Chi-squared test. The association between various factors and overall survival were calculated by the analyses of univariate and multivariate Cox proportional hazard regression model. Kaplan-Meier analysis were used to plot survival cures. P-value <0.05 was considered to be statistically significant. Statistical analyses and graphical presentations were conducted by SPSS 26.0 software and GraphPad Prism Software 8.0.

## Results

### Differential expression and clinical analysis of TFAP2 family in BLCA patients

**Figure 1:**
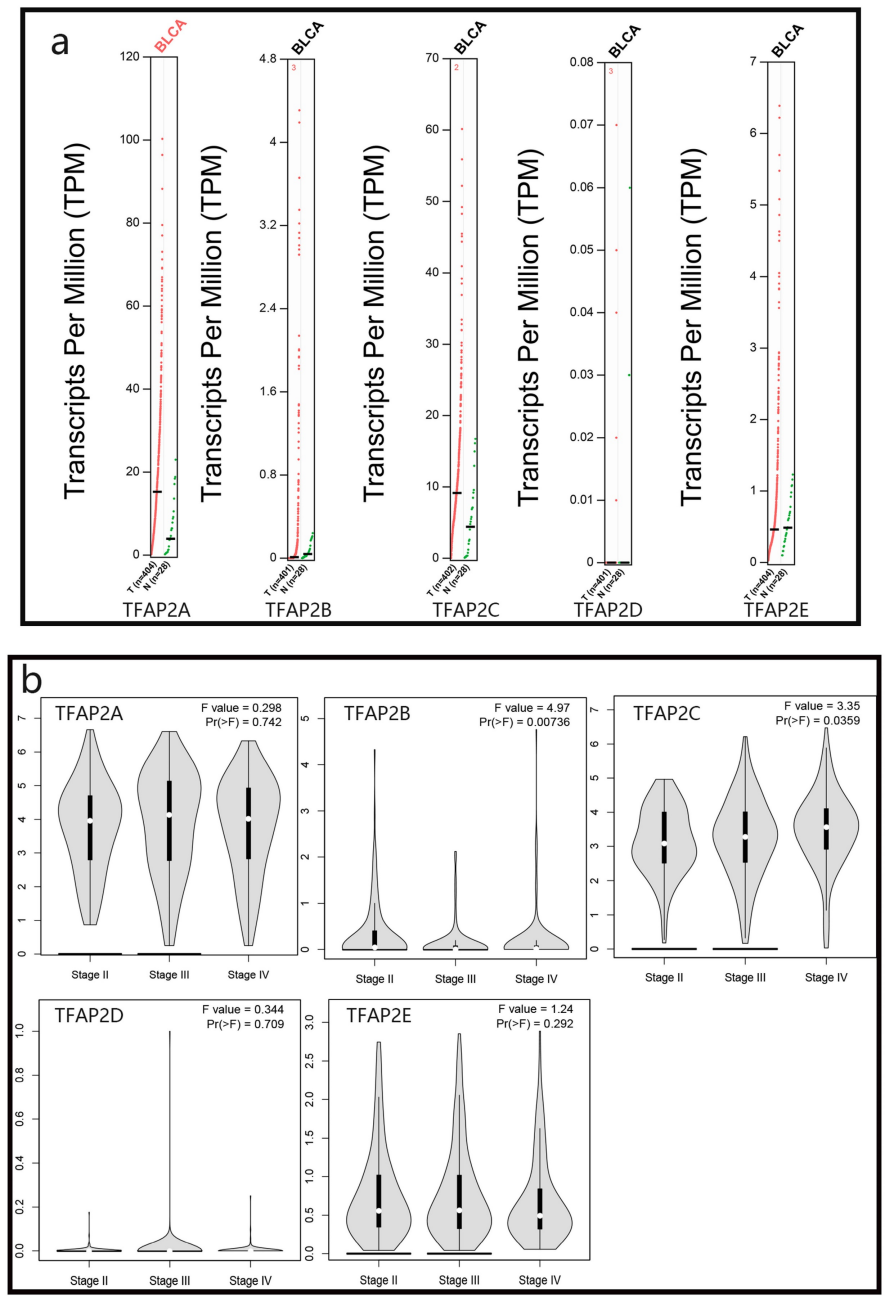
** Expression and clinical analysis of TFAP2s by GEPIA.** (**A**) Scatter diagram demonstrated that the expression levels of TFAP2A was higher in BLCA tissues than in normal tissues. (**B**) The expression of TFAP2C was correlated with the clinical stage of patients with BLCA.

We used GEPIA database to analyze the expression of different TFAP2 family proteins in bladder cancer patients, and found that the expression of TFAP2A, TFAP2B, TFAP2C and TFAP2E in bladder cancer tissues was higher than that in adjacent tissues, although only the expression of TFAP2A was statistically significant (Figure 1a).

We analyzed the relationship between TFAP2 family proteins and the clinical stage of bladder cancer patients, and found that TFAP2C protein was positively correlated with the worse clinical stage of patients, while TFAP2B showed the opposite trend (Figure 1b).

**Figure 2:**
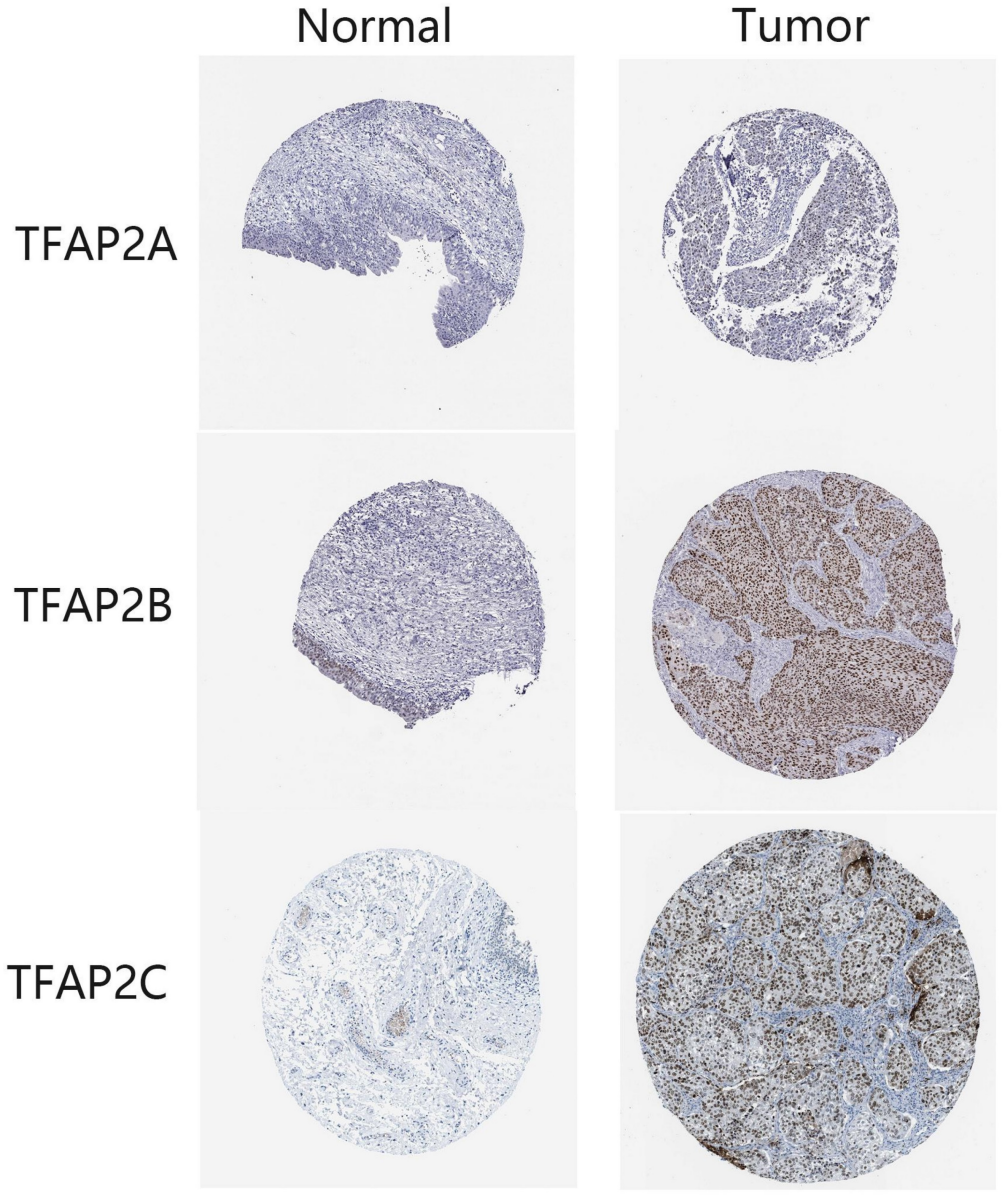
**Expression differences of TFAP2s as detected by immunohistochemistry in BLCA from HPA database.** In normal tissues, TFAP2A, TFAP2B or TFAP2C are hardly expressed or low expressed. Nearly half of BLCA tissues overexpressed TFAP2A, TFAP2B or TFAP2C.

Immunohistochemical results from HPA database showed that the expression of TFAP2A, TFAP2B and TFAP2C in clinical samples of bladder cancer patients was higher than that in normal tissues (Figure 2). TFAP2A and TFAP2C are almost not expressed in normal tissues, while TFAP2B is only low expressed (Table 1).

These data suggested that TFAP2 might play a significant role in the tumorigenesis and progression of BLCA.

### Analysis of the methylation of TFAP2 family and clinical stage in BLCA patients

**Figure 3:**
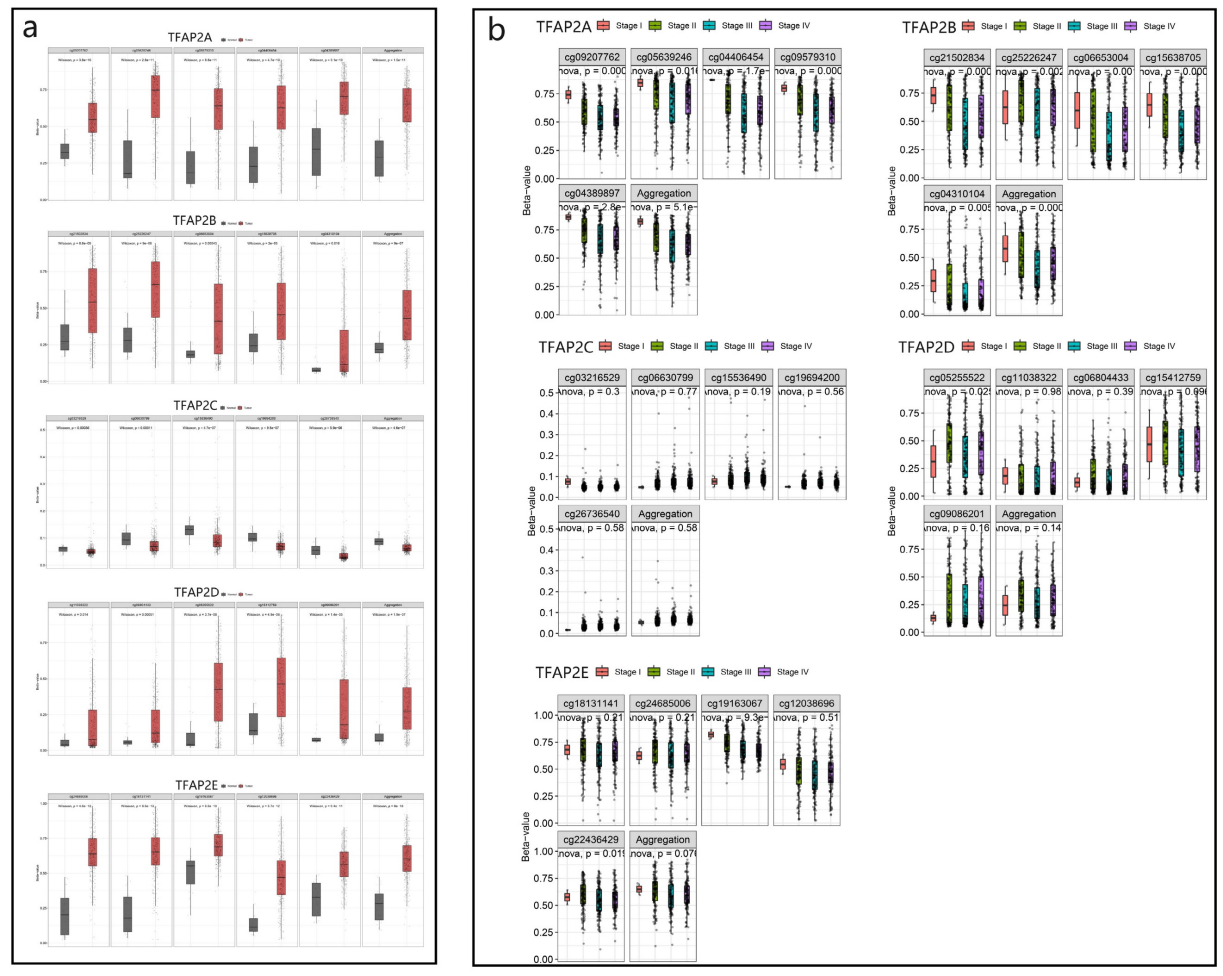
** Methylation and clinical analysis of TFAP2s by SMART.** (a) Scatter diagram demonstrated that the methylation levels of TFAP2 family between BLCA tissues and normal tissues. (b) The expression of TFAP2A and TFAP2B was correlated with the clinical stage of patients with BLCA.

We used SMART database to analyze the methylation of different TFAP2 family in patients with BLCA, and found that the methylation of TFAP2A, TFAP2B, TFAP2D and TFAP2E in bladder cancer tissues was significantly higher than that in adjacent tissues, while TFAP2C showed the opposite trend (Figure 3a).

We analyzed the relationship between TFAP2 family methylation and the clinical stage of BLCA patients, and found that TFAP2A and TFAP2B were correlated with the better clinical stage in BLCA patients (Figure 3b).

### Prognostic value of the mRNA expression of TFAP2 family in BLCA patients

**Figure 4:**
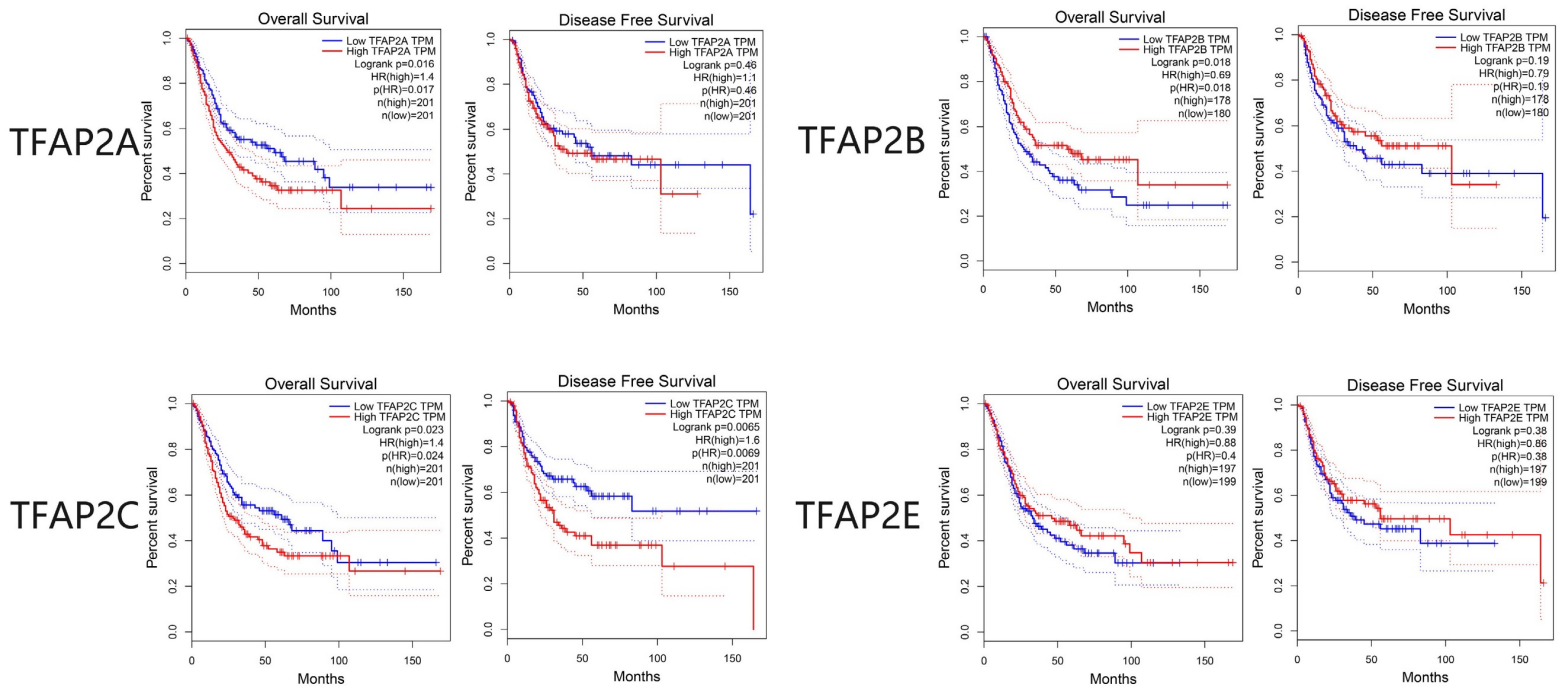
** Prognostic value of the expression of distinct TFAP2s in BLCA (GEPIA).** High transcriptional levels of TFAP2C were significantly associated with short OS and DFS. High expression of TFAP2B is associated with the longer OS of BLCA patients, while TFAP2A shows the opposite trend.

The survival analysis of TFAP2 family genes by GEPIA database showed that TFAP2A, TFAP2B and TFAP2C were significantly related to the survival and prognosis of bladder cancer patients. Disease-free survival (DFS) and overall survival (OS) curves are presented in Figure 4. Similar to clinical staging, BLCA patients with high transcriptional levels of TFAP2C were significantly associated with short OS and DFS. Interestingly, the high expression of TFAP2B is associated with the prolongation of the OS of BLCA patients, while TFAP2A shows the opposite trend.

**Figure 5:**
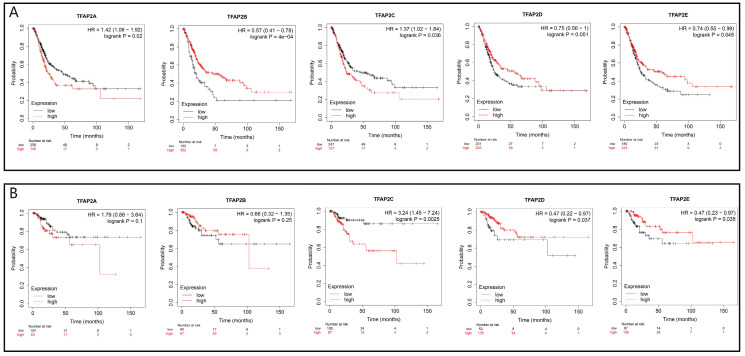
** Prognostic value of the expression of TFAP2s in BLCA (Kaplan-Meier plotter).** (a) Transcriptional levels of TFAP2A and TFAP2C were significantly associated with short OS, while TFAP2B, TFAP2D, TFAP2E were significantly associated with longer OS. (b)High ex-pression of TFAP2D and TFAP2E is associated with the longer RFS of BLCA patients, while TFAP2C shows the opposite trend.

We further analyzed the prognostic value of TFAP2 in BLCA patients with Kaplan Meier plotter (Figure 5). The trend of survival curve of TFAP2A, TFAP2B and TFAP2C in bladder cancer was basically consistent with that of GEPIA. More importantly, Kaplan Meier plotter provided survival analysis data of TFAP2D and TFAP2E. The overexpression of TFAP2D and TFAP2E were associated with the prolongation of sur-vival in BLCA patients.

### Genetic mutation, interaction network and functional cluster analyses of TFAP2 in BLCA patients

**Figure 6:**
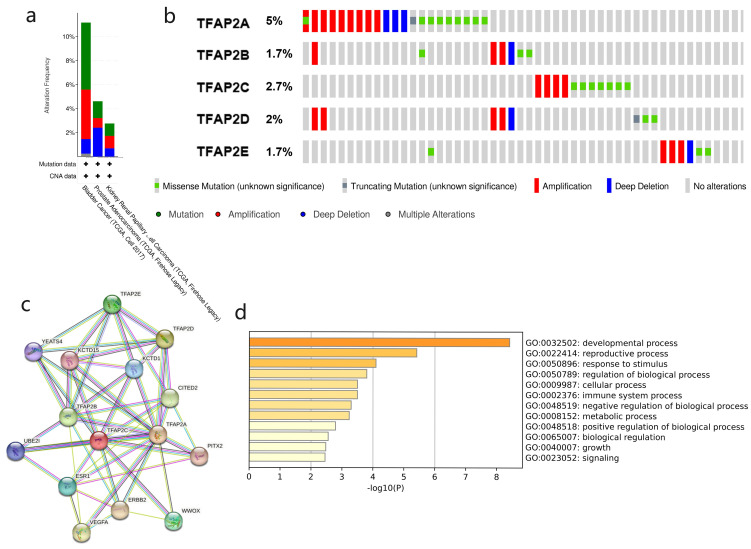
** Gene mutation and PPI network analyses of TFAP2s in BLCA.** (a) Alteration frequency of TFAP2s in BLCA, prostate cancer and kidney renal papillary cell car-cinoma (cBioPortal). (b) Overview of mutations of TFAP2 family proteins in bladder cancer. TFAP2A is the most common mutation gene in bladder cancer (cBioPortal). (c) Protein-protein interaction network of different expressed TFAP2 family proteins (String). (d)KEGG and GO analysis of the proteins above (metascape).

Our study found TFAP2 alterations in bladder cancer is mainly missense mutation with a small amount of truncating mutation. Cbioportal showed that the TFAP2 gene was altered in 46(11.27%, Figure 6a) of 408 patients with BLCA. TFAP2A is the most common gene mutation in bladder cancer patients (5%, Figure 6b), and most of the alterations were amplification (4.17%). Compared with prostate cancer and renal cancer, gene alterations of TFAP2 family are more prevalent in bladder cancer patients.

To explore PPI networks among the TFAP2 family, the 5 proteins were analyzed using the STRING software (Figure 6c). 5 proteins were connected to networks by complex relationships. TFAP2A, TFAP2B, TFAP2C, KCTD15 and KCTD1 showed net-work hubs highly associated with other factors in PPI. Next, we performed PPI enrichment analysis at the Metascape (Figure 6d), which were mainly enriched in the developmental process, reproductive process, response to stimulus, regulation of bio-logical process, cellular process and immune system process, etc.

### Enrichment analysis of TFAP2s functional networks in BLCA

**Figure 7:**
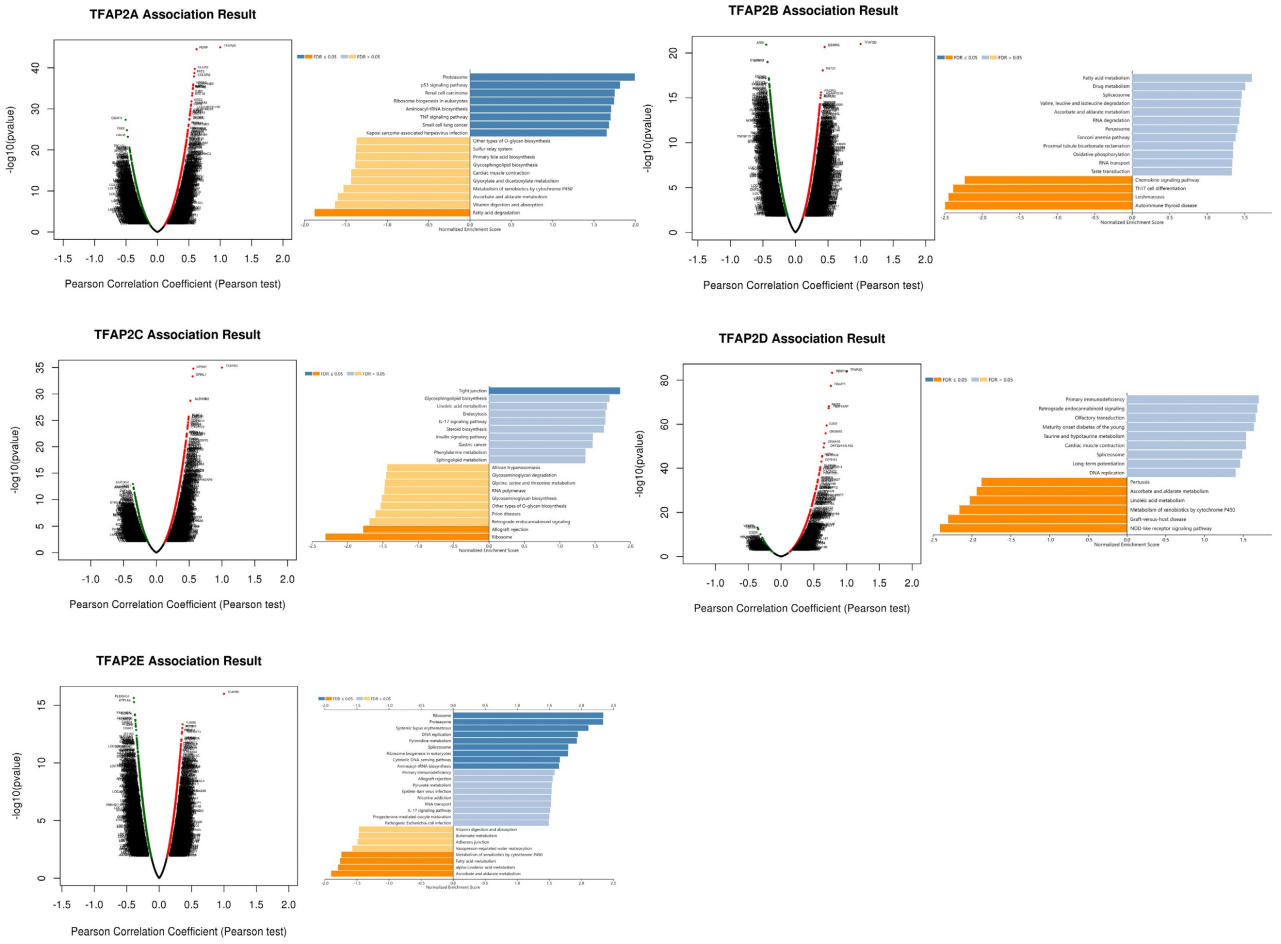
** Enrichment analysis of TFAP2s functional networks in BLCA.** Volcano plot showing the correlations between TFAP2s and genes differentially expressed in BLCA. KEGG analysis by GSEA of the proteins above (LinkInterpreter).

To study the enrichment analysis of TFAP2s functional networks in BLCA, we used the function module of LinkedOmics to study mRNA sequencing data from 408 BLCA patients in the TCGA. According to volcano plot analysis (Figure 7), thousands of genes (dark red dots) showed significant positive relation with TFAP2A, TFAP2B, TFPA2C, TFAP2D and TFAP2E (FDR <0.01). KEGG pathway analysis by GSEA showed enrichment in the p53 signaling pathway, Proteasome, Tight junction, and Fatty acid Metabolism, etc.

### Immune cell infiltration of TFAP2 in BLCA patients

**Figure 8:**
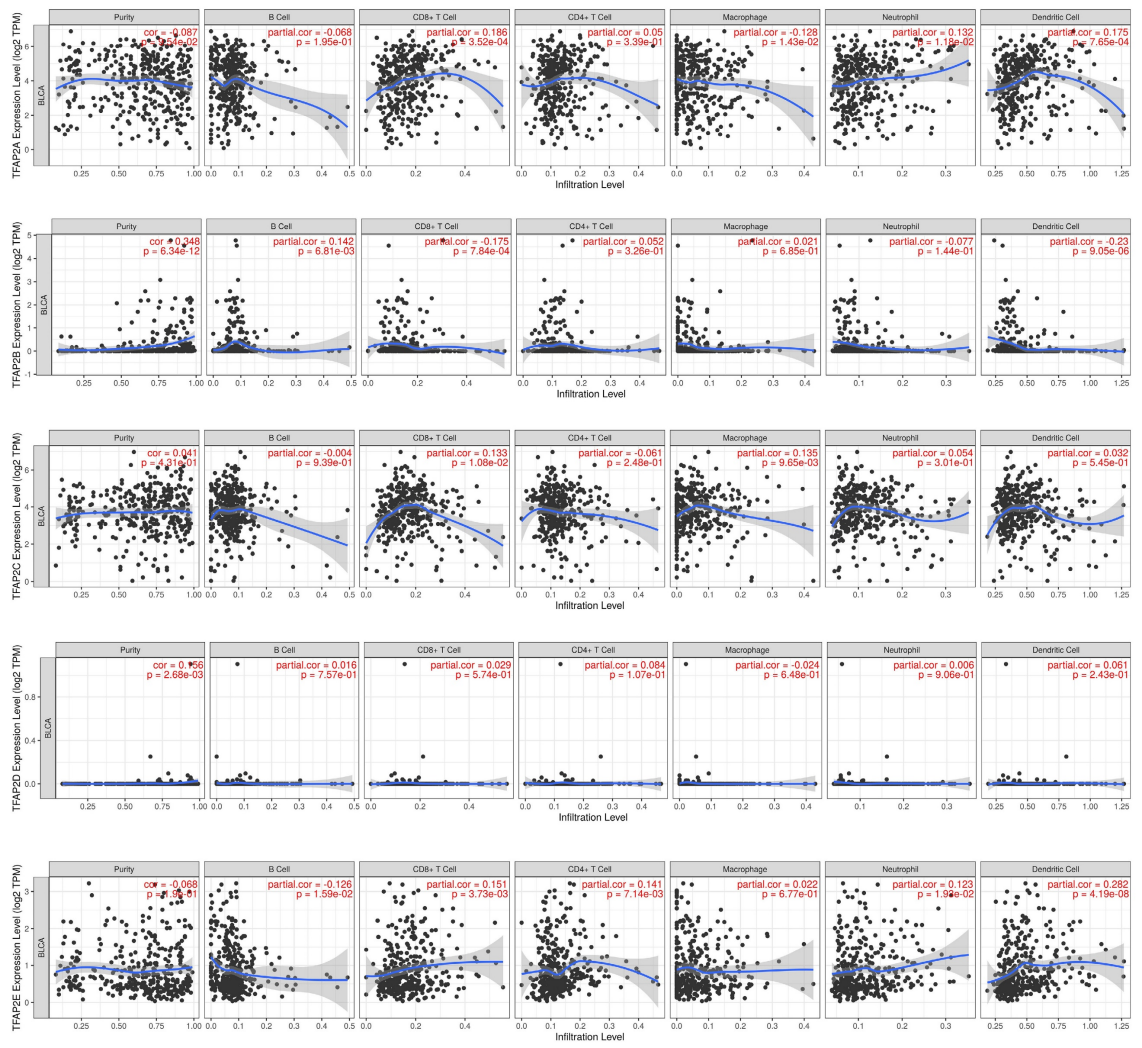
** Correlations between different TFAP2s and immune cell infiltration in BLCA (TIMER).** TFAP2 family was significantly correlated with and the infiltration of six immune cell types in BLCA.

Hence, Spearman's correlation coefficient was applied to analyze the correlation between TFAP2 and immune infiltration level in BLCA patients in TIMER (Figure 8). Our analysis revealed that TFAP2A was in connection with the infiltration of CD8+ T cells, neutrophils, dendritic cells. TFAP2B expression was negatively correlated with CD8+ T cells and dendritic cells. TFAP2C was positively associated with the infiltra-tion of macrophages and CD8+ T cells. With regard to TFAP2E, all host immune cells have a positive correlation in BLCA patients, except macrophages.

**Figure 9:**
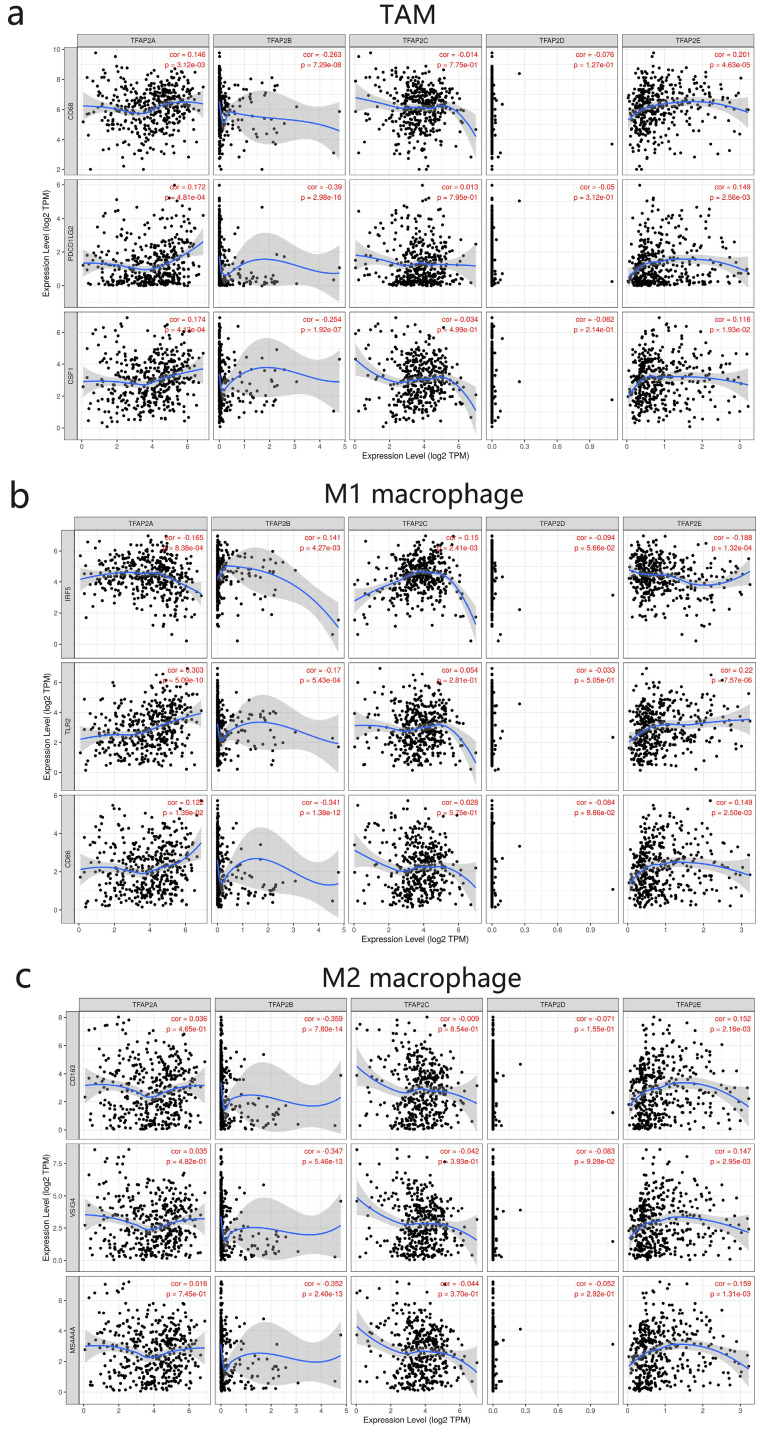
** Correlations between different TFAP2s and TAM in BLCA (TIMER).** TFAP2A, TFAP2B and TFAP2E were significantly correlated with the TAM polarization in BLCA.

We next further explored the link between TFAP2 expression and levels of TAM infiltration based on sets of immunological markers in BLCA using the TIMER (Figure 9). Our study revealed a significant correlation between TFAP2 expression and TAM markers (CD68 PDL2 PDGFB), M1 macrophage markers (IRF5 TLR2 CD86), M2 mac-rophage markers (CD163, VSIG4, MS4A4A). Interestingly, TFAP2A, TFAP2B and TFAP2E expression was correlated with that of the majority of TAM, M1 and M2 mac-rophage markers in BLCA.

**Figure 10:**
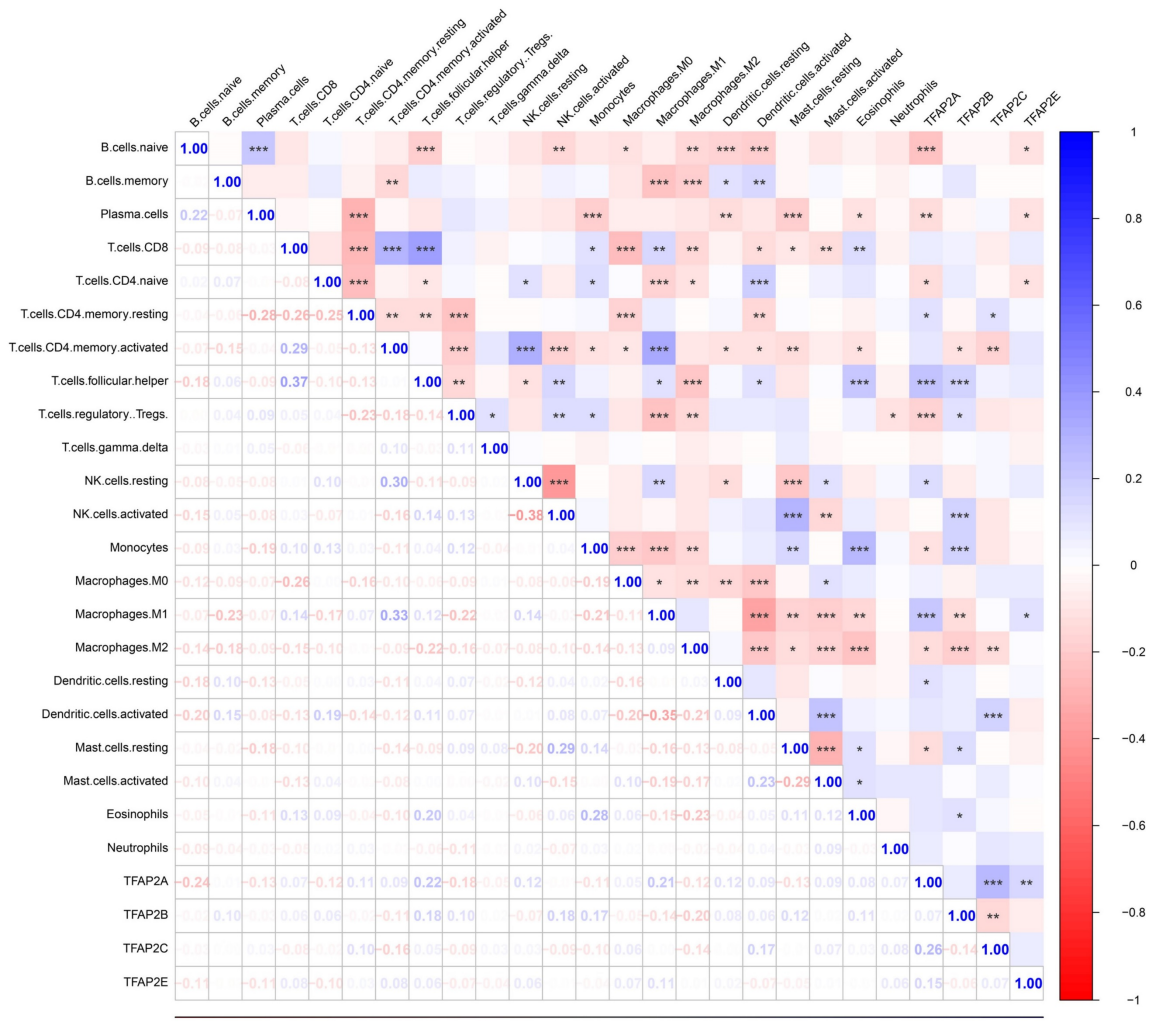
** Correlations between different TFAP2s and immune cell infiltration in BLCA (Cibersort).** Cibersort analysis showed that TFAP2A, TFAP2B, TFAP2C and TFAP2E were significantly correlated with the infiltration of several immune cell types in BLCA. Meanwhile, TFAP2A and TFAP2B have more significant correlation among them.

To further validation, our team employed cibersort analysis to study the correlation between the TFAP2s and immune infiltration (Figure 10). The results showed that TFAP2A, TFAP2B and TFAP2C were significantly associated with the infiltration of various immune cells, which is similar to the outcome above. However, TFAP2A and TFAP2B exhibit more significant correlation with immune reaction. The expression of TFAP2B was positively correlated with the infiltration of monocytes and T cells, while negatively correlated with the polarization of macrophages. Meanwhile, TFAP2A presents a more complex trend in immune cell infiltration, and presents different results in different T cells. Importantly, the expression of TFAP2A was positively correlated with the infiltration of M1 macrophages and negatively correlated with M2 macrophages, suggesting that TFAP2A may be involved in the differentiation of macrophages to M1 types.

### The correlation between TFAP2s and clinicopathological parameters in FFPE cohort

To further verify the relevant data, we collected 240 FFPE BLCA histological specimens, and analyzed the expression of TFAP2s through immunohistochemistry. The correlation between their expression and corresponding clinical data was statistically analyzed using graphpad and spss.

**Figure 11:**
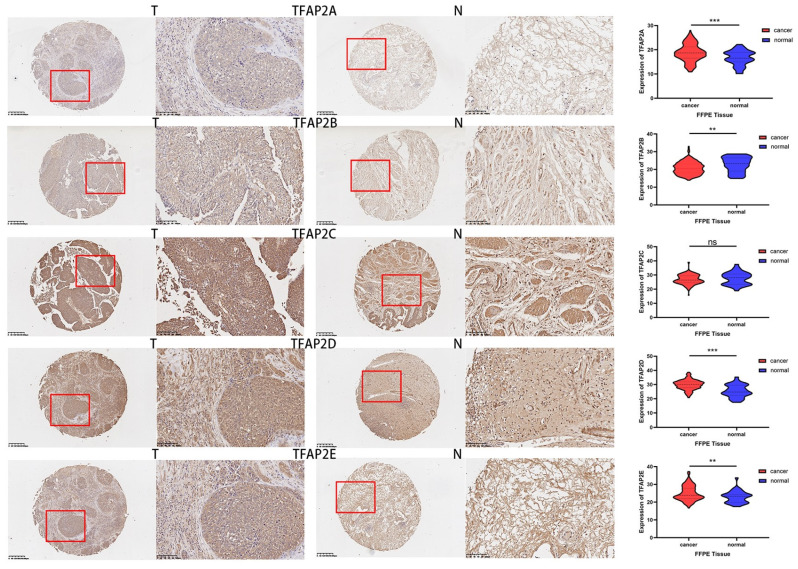
** Expression differences of TFAP2s as detected by immunohistochemistry in FFPE BLCA tissue.** The tumor tissues of 240 patients with bladder cancer and 36 adjacent tissues were made into tissue microarrays, and the expression of TFAP2 family proteins was detected by immunohistochemistry. Graphpad was used for statistical analysis, and the expression of TFAP2A, TFAP2B, TFAP2D and TFAP2E was significantly increased in cancer tissues than that in normal tissues.

Compared with adjacent tissues, the expression of TFAP2A, TFAPB, TFAP2D and TFAP2E in cancer tissue was significantly increased (Figure 11). TFAP2s family showed opposite trend in tumor growth and lymphatic metastasis of BLCA (Table 2). Among them, the expression of TFAP2A, TFAP2B and TFAP2E were significantly higher in T1 and T2 BLCA than in T3 and T4 tumors. However, analysis showed that lymph node metastasis may be significantly associated with high expression of TFAP2A, TFAP2C, and TFAP2E (Table 2). The analysis of tumor differentiation shows that TFAP2A and TFAP2C are closely related to the differentiation of BLCA into basal/squamous cell carcinoma (Table 2).

**Figure 12:**
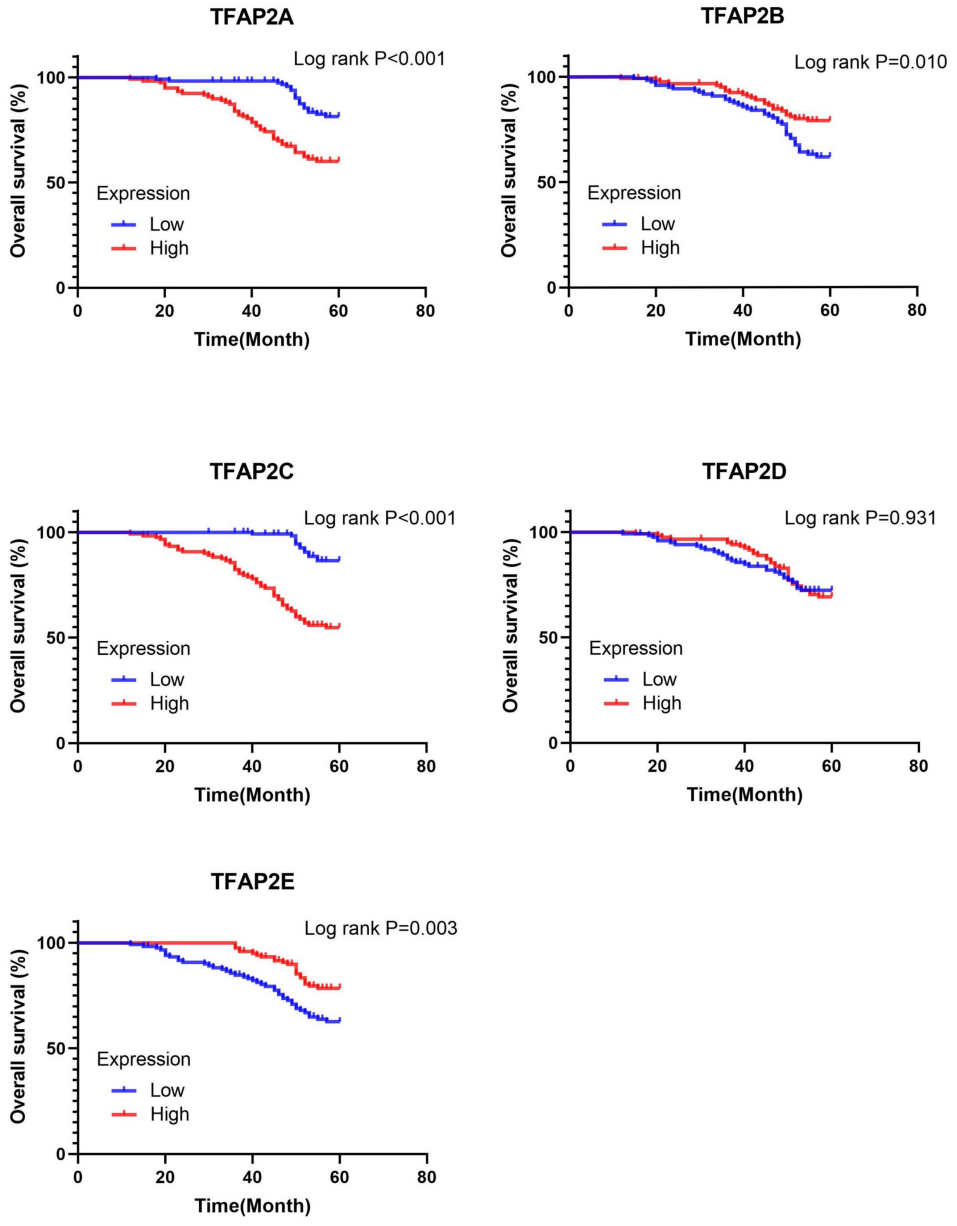
** Prognostic value of the expression of TFAP2s in FFPE Cohort.** TFAP2A and TFAP2C were significantly correlated with poor OS in FFPE cohort. High expression of TFAP2E is associated with increased OS.

Survival analysis (Log-rank test) showed that TFAP2A and TFAP2C may lead to the decline of the overall survival in BLCA patients. While, TFAP2E shows the opposite trend, which is similar to the data from TCGA. However, TFAP2B and TFAP2D did not show significant impact on the survival of BLCA (Figure 12). We used cox regression model to explore the key factors affecting the survival of BLCA. Univariable analysis showed that age, molecular subtype, tumor TNM staging, TFAP2A, TFAP2B, TFAP2C and TFAP2E were important factors affecting the survival of BLCA patients. However, TFAP2D lost its importance in multivariate analysis. Gender, tumor grade, and TFAP2D may not be significantly correlated with tumor survival in the FFPE cohort (Table 3).

## Discussion

Transcription factor activating protein-2(TFAP2) is a family of transcription factors with cell specific binding DNA, which can specifically regulate the expression of target genes. Up to now, five subtypes have been found, which play an important role in regulating cell proliferation, differentiation, apoptosis and mammalian embryonic development. In recent years, its role in tumorigenesis and cancer development has also received increasing attention. Numerous studies have shown that the regulatory effect of TFAP2 may be bidirectional, that is, tumor inhibition or tumor promotion, which is related to tissue specificity, time sequence and differences among various subtypes.

TFAP2A functions as a tumor suppressor and influences response to therapy in several cancer types. Study in colorectal cancer showed that loss of TFAP2A delayed progression through S-phase into G2-M and decreased phosphorylation of AKT, which were mediated through regulation of TGM2. Importantly, activation of AKT leading to resistance to the PI3K inhibitor, Buparlisib, after degradation of TFAP2A 13. Interestingly, a recent study showed that TFAP2A drives melanoma metastasis by upregulating E2F pathway genes including EZH2 through inhibition of the NuRD repression complex, serving as a biomarker to predict responsiveness to EZH2 inhibitors 14.

In RT-112 cell line (grade II bladder cancer), TFAP2A and WWOX overexpression induced apoptosis but decreased cell viability, adhesion, matrix metalloproteinase-2 activity, overall number of cultured colonies and migration rate, inducing the tumor progress 9. However, the researchers found that the appeal situation changed in high-level bladder cancer 15. WWOX and TFAP2A demonstrate tumor suppressor synergism in high-grade bladder cancer, similar to intermediate grade. However, WWOX does not appear to guide oncogenic TFAP2C, which should be further investigated.

In recent years, studies on TFAP2C have found that it has similar DNA binding sites, similar anti-tumor effects and mechanisms with TFAP2A. Recent study in BLCA revealed that TFAP2C knockdown affected the activation levels of EGFR and NF-κB and enhanced the anti-tumor effects of cisplatin in vivo and in vitro 16. However, our study shows that the expression of TFAP2C is positively correlated with the clinical stage of bladder cancer, and patients with high expression of TFAP2C have a shorter survival period.

Our study found that the expression of TFAP2A was increased in BLCA, both at the transcriptional level and protein level. Also, the methylation level of TFAP2A was negatively correlated with the clinical stage of bladder cancer. Moreover, increased expression of TFAP2A and TFAP2C was associated with shortened survival in patients with BLCA. These evidences suggest that TFAP2A and TFAP2C may play an oncogene in BLCA and their increased expression may be related to the occurrence and progression of BLCA. Further verification is needed to determine whether TFAP2A and TFAP2C plays an oncogenic role or not in BLCA.

TFAP2B has been proven to promote the progression of non-small cell lung cancer and thyroid cancer through VEGF/PEDF and COX-2 signaling pathway. But our data from FFPE sample showed that TFAP2B is related to the earlier T stage of bladder cancer, but not to lymph node metastasis, which suggests that TFAP2B may be an inhibitor of bladder cancer growth. Moreover, our survival analysis showed that high expression of TFAP2B was positively correlated with prolongation of survival in BLCA patients, which was contrary to the results of other members of other genes in TFAP2 family. A whole genome methylation sequencing results of bladder cancer showed that the methylation level of TFAP2B in high-level bladder cancer was significantly higher 17, which is consistent with our finding. These results suggest that TFAP2B may play a role of tumor suppression in bladder cancer. However, further study found that the hypermethylation levels of TFAP2A and TFAP2B were positively correlated with earlier clinical stages, which needs further research.

At present, the research on TFAP2D and TFAP2E in tumor is rare, compared to other genes in TFAP2 family. A study in prostate cancer revealed that TFAP2D staining was significantly linked to advanced tumor stage, high classical and quantitative Gleason grade, lymph node metastasis, and a positive surgical margin 18. Hypermethylation of TFAP2E has been reported to be associated with chemoresistance to 5‑fluorouracil (5‑FU) in gastric cancer (GC) 19. But a recent large-scale multicenter cohort study indicated that TFAP2E methylation and expression may not play a major role in predicting response to 5-FU-based chemotherapy in patients with colorectal cancer 20. In our study, TFAP2E showed the opposite trend in the tumor growth and lymph node metastasis of bladder cancer. But in general, TFAP2E and TFAP2D has a better prognosis in bladder cancer. However, less study on TFAP2D and TFAP2E was carried out in bladder cancer, and its specific role and mechanism need further research.

Further genetic analysis indicated frequent genetic alterations in the TFAP2 family that are differentially expressed in BLCA. We used string to analyze and predict the proteins interacting with TFAP2 family. The functions of these proteins are mainly focused on developmental process, reproductive process and immune process, etc.

A recent study shows that TFAP2A can regulate the function of tumor related macrophages in colorectal cancer 21. We observed a strong correlation between TFAP2 and M1/M2 macrophage markers including CD68, PDL1, PDGFB, IRF5, TLR2, CD86, CD163, VSIG4 and MS4A4A (Figure 7). This suggests that TFAP2A, TFAP2B and TFAP2E play a role in regulating TAM polarization. Moreover, our study showed that the expression of TFAP2 family was significantly correlated with and the infiltration of six immune cell types, including B cells, CD4+ T cells, CD8+ T cells, Neutrophils, Macrophages, and Dendritic cells, suggesting that TFAP2A, TFAP2B, TFAP2C and TFAP2E may also reflect the immune status besides the disease prognosis. Our study might provide detailed immunization information to assist in the design of new immunotherapies.

TFAP2s family is an important transcription factor, and a large number of down-stream proteins are regulated by TFAP2s family. GSEA analyses showed that the functional networks of TFAP2s was related to the occurrence and development of several tumors, like renal cell carcinoma, small cell lung cancer, and gastric cancer, etc. Interestingly, TFAP2s participates in multiple metabolic pathways in the body, including fatty acid metabolism, drug metabolism, ascorbate and aldarate metabolism, linoleic acid metabolism and phenylalanine metabolism, etc. Moreover, these pathways are closely related to the tumor growth, drug resistance and immunotherapy in various cancers 22, 23.In summary, TFAP2 family might be an important regulator of immune cell infiltration and a valuable prognostic biomarker in patients with BLCA.

## Figures and Tables

**Table 1:** Detection of TFAP2 gene by immunohistochemistry in bladder cancer from HPA database

Gene	Normal tissue	Bladder cancer		
Staining intensity		Low	Medium	High
TFAP2A	Not detected	3/11	1/11	
TFAP2B	Low	2/12	1/12	5/12
TFAP2C	**Not detected**	**2/11**		**1/11**

**Table 2:** Relationship Between TFAP2s and Clinicopathologic Characteristics in Patients with Bladder Cancer from FFPE Cohort

Parameter	Chi-squared test Mean difference and P value				
	TFAP2A	TFAP2B	TFAP2C	TFAP2D	TFAP2E
Age (<65 vs ≥65) (126 vs 114)	*p*=0.685	*p* =0.941	*p* =0.142	*p* = 0.924	*p* =0.225
Gender (Female vs Male) (36 vs 204)	*p* =0.150	*p* =0.867	*p* =0.926	*p* =0.899	*p* =0.498
Grade (Low vs High) (20 vs 220)	*p* =0.473	*p* =0.308	*p* =0.919	*p* =0.477	*p* =0.669
Molecular subtype (Basal vs Luminal) (92 vs 148)	3.040 ***p* <0.001**	*p* =0.219	2.528 ***p* <0.001**	*p* =0.381	*p* =0.362
T stage (T1-2 vs T3-4) (164 vs 76)	1.741 ***p* =0.001**	1.231 ***p* =0.009**	*p* =0.540	*P*=0.931	3.003 ***p* <0.001**
N stage (N1 vs N0) (148 vs 92)	1.773 ***p* <0.001**	*p* =0.945	1.143 ***p* =0.004**	*p* =0.069	1.220 ***p* =0.019**

**Table 3:** Univariate and Multivariate Cox Regression Analysis for Overall Survival of Patients with Bladder Cancer

	Univariate analyses		Multivariate analyses	
	HR (95% CI)	p	HR (95% CI)	p
Age (<65 vs ≥65) (126 vs 114)	2.247 (1.362 ~3.707)	0.002	2.544 (1.512 ~ 4.282)	<0.001
Gender (Female vs Male) (36 vs 204)		0.505		
Grade (Low vs High) (20 vs 220)		0.108		
Molecular subtype (Basal vs Luminal) (92 vs 148)	3.019 (1.837 ~ 4.961)	**<0.001**	2.563 (1.351 ~ 4.860)	**0.004**
T stage (T3-4 vs T1-2) (76 vs 164)	2.643 (1.623 ~ 4.303)	**<0.001**	4.292 (2.145 ~ 8.590)	**<0.001**
N stage (N1 vs N0) (148 vs 92)	3.814 (1.944 ~ 7.484)	**<0.001**	4.026 (1.902 ~ 8.520)	**<0.001**
TFAP2A (High vs Low) (120 vs 120)	2.872 (1.694 ~ 4.869)	**<0.001**	2.276 (1.131 ~ 4.582)	**0.021**
TFAP2B (High vs Low) (120 vs 120)	0.522 (0.315 ~ 0.864)	**0.012**		0.413
TFAP2C (High vs Low) (120 vs 120)	3.882 (2.699 ~ 8.829)	**<0.001**	5.256 (2.707 ~ 10.206)	**<0.001**
TFAP2D (High vs Low) (120 vs 120)		0.931		
TFAP2E (High vs Low) (120 vs 120)	0.417 (0.249 ~ 0.698)	**0.001**	0.207 (0.111 ~ 0.387)	**<0.001**

## Abbreviations

BLCA: bladder cancer
TFAP2: transcription factor activating protein-2
FFPE: formalin-fixed paraffin-embedded
HPA: human protein atlas
GEPIA: gene expression profiling interactive analysis
SMART: shiny methylation analysis resource tool
TAM: tumour-associated macrophages
TCGA: the cancer genome atlas
GEO: gene expression omnibus
GO: gene ontology
KEGG: kyoto encyclopedia of genes and genomes
CPTAC: clinical prote-omics tumor analysis consortium
OS: overall survival
DFS: disease-free survival
PPI: protein-protein interaction
GSEA: gene set enrichment analysis
